# Auriculotherapy used to manage orthodontic pain: a randomized controlled pilot study

**DOI:** 10.1590/2177-6709.26.6.e2119381.oar

**Published:** 2021-12-17

**Authors:** Emanuela SERRITELLA, Alessandra IMPELLIZZERI, Aldo LIGUORI, Gabriella GALLUCCIO

**Affiliations:** 1 “Sapienza” University of Rome, Department of Oral and Maxillofacial Sciences (Rome, Italy).; 2 “Paracelso Institute” of Rome, Moral Institution of the Ministry of Health, (Rome, Italy).

**Keywords:** Pain, Fixed orthodontic appliance, Auriculotherapy, Acupuncture

## Abstract

**Introduction:**

Several methods are commonly used to decrease orthodontic pain, but versatile tools and standardized protocols are still lacking.

**Objective:**

In response to the need for alternatives to conventional analgesic methods, this study evaluates the analgesic effects of auriculotherapy (AT) during the first three months of fixed orthodontic treatment.

**Methods:**

A sample of 36 subjects was selected, with patients randomly allocated into two homogeneous groups, Study Group (SG) and Control Group (CG), depending on the application/non-application of AT. Patients rated their pain scores monthly from 0 to 10, on visual analogue scales (VAS) at the time of bonding (T_0_) and again at two appliance adjustments (T_1_ and T_2_). At each of these treatment phases, VAS was applied in six different time moments (TM): immediately before, immediately after, after 4 hours, after 8 hours, after 24 hours, and after 72h hours. Descriptive statistical analysis, a Student’s t-test, and a Chi-square test were applied to the collected data (statistical significance for *p*< 0.05).

**Results:**

SG patients reported lower pain levels than CG patients, both at T_0_, T_1_ and T_2_. Moreover, average pain intensity values were lower in the SG for all TM analyzed, with the *t*-test significant (*p*< 0.05) for most TMs.

**Conclusion:**

AT was effective in the pain treatment of patients with fixed orthodontic appliances. Further studies are needed with a sham control group to confirm the validity of these results.

## RESUMO

**Introdução:**

Vários métodos são comumente usados para diminuir a dor proveniente do tratamento ortodôntico, mas ainda faltam ferramentas versáteis e com protocolos padronizados.

**Objetivo:**

Em resposta à necessidade de alternativas aos métodos analgésicos convencionais, o presente estudo avalia os efeitos analgésicos da auriculoterapia (AT) durante os primeiros três meses de tratamento ortodôntico com aparelhos fixos.

**Métodos:**

Foi selecionada uma amostra de 36 indivíduos, e os pacientes foram alocados aleatoriamente em dois grupos homogêneos: Grupo Experimental (GE) e Grupo Controle (GC), com aplicação / não aplicação da AT, respectivamente. Os pacientes registraram seus escores de dor de 0 a 10, mensalmente, em escalas visuais analógicas (EVA) logo após a colagem do aparelho (T_0_) e novamente em duas consultas de manutenção do aparelho (T_1_ e T_2_). Em cada uma dessas fases do tratamento, a EVA foi aplicada em seis momentos diferentes (MT): antes, imediatamente após, após 4 horas, após 8 horas, após 24 horas e após 72 horas. Uma análise estatística descritiva, o teste *t* de Student e o teste do Qui-quadrado foram aplicados aos dados coletados (significância estatística para *p* <0,05).

**Resultados:**

Os pacientes do GE relataram níveis de dor mais baixos do que os pacientes do GC, tanto em T_0_ quanto em T_1_ e T_2_. Além disso, os valores médios de intensidade da dor foram menores no GE em todos os MTs analisados, com o teste *t* significativo (*p* <0,05) para a maioria das MTs.

**Conclusão:**

A AT foi eficaz no tratamento da dor em pacientes com aparelhos ortodônticos fixos. Mais estudos são necessários, com um grupo de controle placebo, para comprovar a validade desses resultados.

## INTRODUCTION

Orthodontic therapies, like most dental procedures, cause emotional stress to the patient and are often associated with pain that at times may even be intense.^1^ Orthodontic pain may be perceived during all orthodontic procedures, but several studies have shown that fixed orthodontic appliances cause more intense pain than removable or functional ones.^2^ ^,^ ^3^ Different methods for orthodontic pain management have been studied, including the use of pharmacological and mechanical therapies, laser therapies, and behavioral strategies.^4^ ^,^ ^5^ While these therapeutic strategies have proven useful in the management of pain during treatment, orthodontists generally do not prioritize pain management; the lack of standardized and efficacy-proven pain protocols reflects this point.

The use of unconventional therapeutic protocols as alternatives to support conventional medical methods is much-discussed in medical and scientific literature.^6^ Among Non-Conventional Medicines (NCM), Traditional Chinese Medicine (TCM) is the most widespread globally, as well as the most systematic, since it is organized around several key principles that have been preserved for centuries. Acupuncture, one of its main branches, is a versatile therapeutic tool that has been applied in various medical areas, including Dentistry.^7^ ^,^ ^8^ Several studies indicate that acupuncture could supplement conventional dental treatment methods, in particular for treatment of dental anxiety, gag reflex, temporomandibular dysfunction, facial/neck pain, and headache.^9^ ^,^ ^10^ Acupuncture for analgesic purposes is of particular interest. Although many of acupuncture’s physiological and neurological mechanisms are still unknown, the efficacy of therapeutic acupuncture for pain therapy has been well established.^11^ ^,^ ^12^ While several studies support the use of acupuncture for the management of dental pain,^13^ ^,^ ^14^ the most investigated field for the use of acupuncture in pain management is facial and cranial-cervico-mandibular pain.^15^ ^,^ ^16^ Conversely, acupuncture has not been extensively studied for the treatment of orthodontic pain, though there is some evidence of the effectiveness of somatic acupuncture in this area.^17^ ^,^ ^18^

On the basis of this preliminary scientific evidence, it was decided to undertake an experimental study to evaluate the severity and evolution of pain in the early stages of fixed orthodontic therapy and to verify the efficacy of analgesic acupuncture in patients undergoing these kinds of therapies. The study was carried out by applying an acupuncture method not yet studied in the management of orthodontic pain: auriculotherapy (AT), which involves the application of *Vaccaria* seeds to specific auricular acupoints. Compared to other acupuncture methods involving the insertion of needles at the cutaneous level, auriculotherapy offers the additional advantage of being well-received by pediatric subjects. The study results demonstrate this technique to be an effective treatment for a variety of types of pain, both acute and chronic.^19^ ^,^ ^20^

This research aimed to analyze the efficacy of auriculotherapy in the control of pain associated with the use of fixed orthodontic appliances, and to propose a therapeutic protocol effective for reducing orthodontic pain.

## MATERIAL AND METHODS

A consecutive series of eligible patients was selected from a university hospital ward, with no discrimination with regards to gender or age, between May 2018 and May 2019. All patients included in the study were undergoing a conventional fixed multibracket orthodontic therapy, applied to one or both dental arches. Patients with intellectual disabilities, chronic and/or metabolic diseases, as well as those undergoing analgesic drug therapy or who had already begun or previously undergone orthodontic treatment, were excluded. All patients were informed about the study, including its aims and the potential risks, and signed an informed consent form in advance. The study was approved by the Institutional Ethics Committee, Department of Oral and Maxillofacial Sciences, Sapienza University, Rome, Italy (#53/2018 - 0000711). 

Selected patients were randomly allocated into two groups, depending on the application/non-application of auriculotherapy during the first three months of orthodontic treatment:

» Study Group (SG): auriculotherapy.
» Control Group (CG): no auriculotherapy.

During the session for placement of the fixed orthodontic appliances, a *pain assessment card* was distributed to all participants, which was then returned to the medical staff, once fully completed.

All participants were asked to indicate their pain perception in the oral cavity and the maxillofacial region during the initial period of orthodontic treatment, at the following three time intervals:

» Start of therapy (T_0_): time at which the bonding of one or both dental arches occurred.
» First adjustment (T_1_): one month after T_0_. Time at which the orthodontic appliance was adjusted, by changing the archwire of one or both dental arches.
» Second adjustment (T_2_): one month after t1. Time at which the orthodontic appliance was adjusted, by changing the archwire of one or both dental arches.

A Visual Analogue Scale (VAS) was used as a tool of pain self-assessment. The scale is represented as a straight line of 10 cm between two poles: “no pain” (0) and “maximum pain” (10). Study participants were instructed to indicate the value of pain experienced during all three times intervals by plotting a mark on the scale. Patients’ pain sensations were arbitrarily divided into five categories, based on the value given on the VAS scale: no pain (0), mild (1-3), medium (3-5), severe (5-7), very severe (> 7).

Both at the beginning of therapy and in the following two adjustments (T_0_, T_1_ and T_2_), the VAS scale was used to quantify the sensation of pain immediately before, immediately after, after 4 hours, after 8 hours, after 24 hours, and after 72 hours. Each patient therefore completed six pain assessments at each of the above-mentioned time intervals (T_0_, T_1_and T_2_) during the first three months of therapy. 

In addition to the values related to pain perception, epidemiological data were collected, as well as data on the pathology and type of therapy performed: age, gender (male, female), malocclusion (Class I, Class II, Class III), and dental arch treated (upper, lower, both). 

### AURICULOTHERAPY TREATMENT PROTOCOL

**Figure 1:**
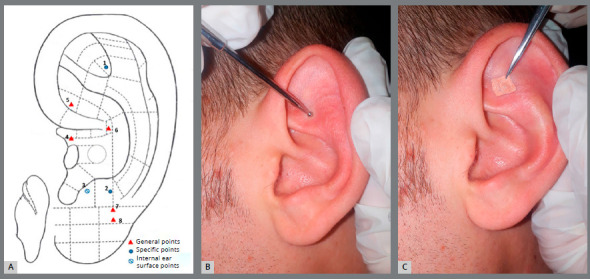
A) Map of auricular points used: (1) Shenmen; (2) Occiput; (3) Subcortex; (4) Mouth; (5) Large Intestine; (6) Stomach; (7) Lower jaw; (8) Upper jaw. Example of (B) sensitive points research and of (C) *Vaccaria* seed application.

A treatment protocol was developed according to the standardized auriculotherapy treatment methods used in traditional Chinese medicine (TCM), with the selection of application points based on TCM principles.^21^ We used both specific pain points (Shenmen, Subcortex, Occiput) and points defined as general that pertained to the topographic location of the pain sensation (Mouth, Large Intestine and Lower Jaw for the lower arch, Stomach and Upper Jaw for the upper arch) (Fig 1A). The protocol was elaborated and defined in collaboration with the Paracelso Institute of Rome (Moral Institution of the Ministry of Health m.d. 15 April 1996; China-Italy Center of Traditional Chinese Medicine) and was performed by an experienced acupuncturist licensed in TCM.

### PROCEDURE

The treatment was performed on a single auricle at each time interval. At the beginning of orthodontic therapy (T_0_), the side for auriculotherapy application was selected. Side selection was based on the patient’s gender, with application on the right side for females and left side for males. The sides treated were then alternated at the following two adjustments (T_1_ and T_2_). 

Treatment began after the ear surface was cleaned with a cotton wool pad soaked in ethyl alcohol and then dried. Three points for pain control were selected: the “Mouth” point and the points relating to the dental arch on which the fixed orthodontic appliance was applied. When both dental arches were bonded at the same time, all protocol points were selected. An auriculotherapy speculum was used to identify sensitive or reactive points, through palpation of the ear surface. Once a reactive or sensitive area was located, the speculum was pressed deeper so as to leave a mark on the skin corresponding to the point to be treated. A *Vaccaria* seed was applied immediately afterwards and fixed to the skin with a bandage (Fig.1B, 1C). This procedure was repeated for all the points to be treated. The auriculotherapy procedure started within 5 minutes of the end of the clinical bonding procedures (T_0_) and the following adjustments (T_1_ and T_2_). The *Vaccaria* seeds remained in place for three days after each application, and then were removed by the patient; the points where the seeds were placed were subjected to intermittent pressure for about one minute, 3 to 5 times a day, for the duration of the three days. This technique was taught to all participants, and it was confirmed that patients had correctly carried it out at each time interval. The alternate ear was treated in the same way after the subsequent adjustments. 

Since data regarding the application of auriculotherapy in orthodontic pain was not available from other clinical studies, patients were recruited using convenience sampling.

All patients were randomly assigned to the control (CG) or study group (SG) at a rate of 1:1 allocation. The randomization was performed using a multiplicative congruential generator of pseudo-randomized numbers (Lehmer RNG). Patients were enrolled sequentially, based on the order produced by the generator. To increase the accuracy of the study, randomization continued until we reached the same number of patients in both groups; the final sample size was made up of 36 subjects (18 in each treatment group).

Given the characteristics of the treatment, participants and investigators were not blinded to treatment allocation. The statistician was not involved in the randomization process and analyzed data without assess to information about allocation.

The primary outcome of interest was the pain level and its variation over the three time intervals (T_0_, T_1_ and T_2_) in both groups, for all the time moments analyzed. The secondary outcome was pain level distribution according to the epidemiological data (age, gender, malocclusion, and treated dental arch). 

### STATISTICAL ANALYSIS

All obtained data were examined using SAS software. Descriptive statistical analysis involved calculation of the following quantitative characteristics: standard deviation, mean, median, minimum, and maximum. This analysis was carried out for both epidemiological characteristics: those related to the therapy and those concerning pain intensity and its trends over time. Performance of a Student’s *t*-test allowed for identification of significant differences between the average pain levels of the study group and the control group; a Chi-square test was performed to determine significant differences between the average pain levels according to gender, age, malocclusion, and treated dental arch *(*statistical significance for *p*< 0.05*).*

## RESULTS

**Figure 2:**
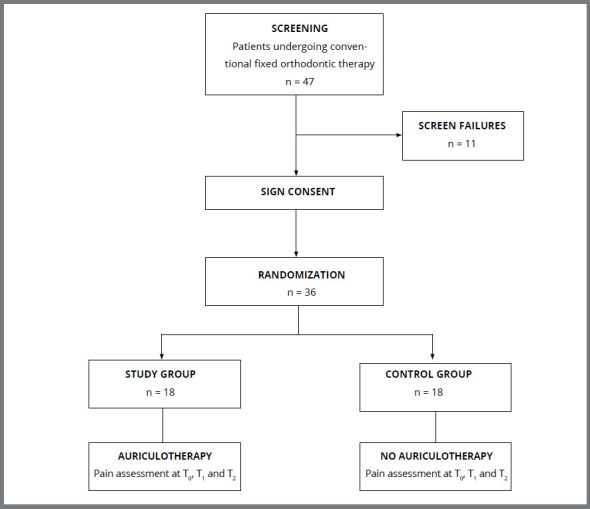
Flow chart of patient enrollment and interventions.

The study sample was comprised of 36 Caucasian subjects, 14 male and 22 female, with a mean age of 19.5 years (range: 13-54 years) (Fig 2). Table 1 presents the characteristics of the whole sample with regard to demographic data, pathology, and type of therapy performed.

**Table 1:** Participants’ epidemiological data.

	Study group (SG)	Control group (CG)	TOTAL (SG + CG)
Age			
MEAN ± S.D.	20.89 ± 6.67	18.05 ± 10.10	19.47 ± 8.48
Gender			
MALE (n, %)	8 (44.4%)	6 (33.3%)	14 (38.9%)
FEMALE (n, %)	10 (55.6%)	12 (66.7%)	22 (61.1%)
Malocclusion			
Class I	8	4	12
Class II	7	10	17
Class III	3	4	7
Treated dental arch			
Upper	14	14	28
Lower	4	4	8
Both	0	0	0

**Figure 3:**
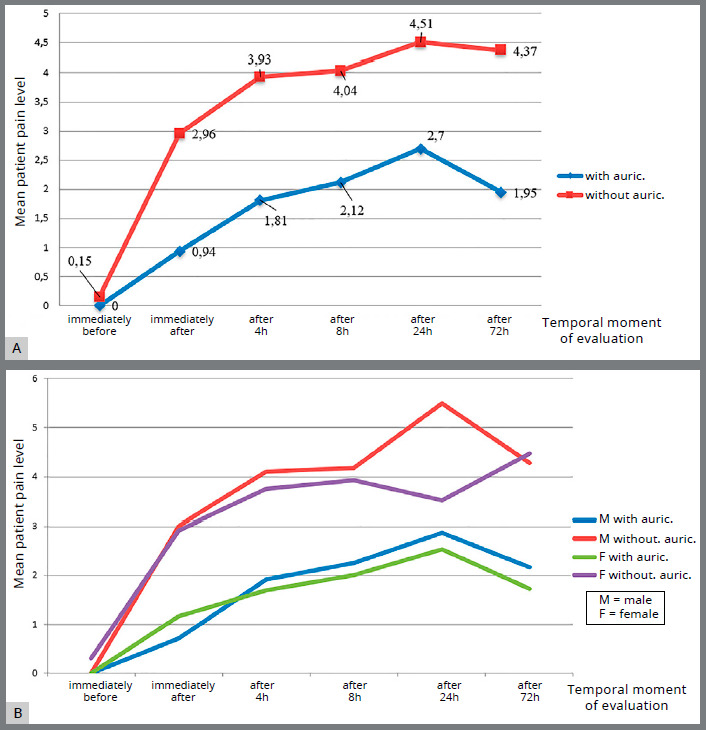
Mean pain levels over time in patients with and without auriculotherapy**(**A), and based on patient’s gender **(**B).

SG patients reported lower pain values​​ than CG patients. These values were lower, on average, for all the time intervals analyzed (T_0_, T_1_ and T_2_). Figure 3 shows the quantitative pain results (VAS scale) in terms of mean values ​​of pain perception for both groups (SG and CG) across all three time intervals analyzed (T_0_, T_1_ and T_2_), with indication of whether or not patients underwent auriculotherapy treatment (Fig 3A), and divided according to the gender (M and F) of the patients (Fig 3B). 

The values ​​reported by male patients were higher, on average, than those reported by females for all the time intervals considered, with the exception of the moments “immediately after” for SG patients and “immediately before” and “after 72 hours” for CG patients (Table 2 and Fig 3B).

**Table 2:** Average pain levels over time based on patient’s gender (mean values for the three time intervals considered: T_0_, T_1_ and T_2_).

SG - WITH AURICULOTHERAPY (mean ± S.D.)						
Gender	Immediately before	Immediately after	After 4h	After 8h	After 24h	After 72h
M	0	0.71 ± 0.69	1.92 ± 1.84	2.25 ± 2.00	2.87 ± 2.4	2.17 ± 2.10
F	0	1.17 ± 1.08	1.70 ± 1.53	2.00 ± 1.62	2.53 ± 2.09	1.73 ± 1.70
CG - WITHOUT AURICULOTHERAPY (mean ± S.D.)						
Gender	Immediately before	Immediately after	After 4h	After 8h	After 24h	After 72h
M	0	3.00 ± 2.30	4.11 ± 1.84	4.17 ± 2.06	5.50 ± 2.20	4.28 ± 1.77
F	0.30 ± 0.67	2.92 ± 2.29	3.75 ± 2.09	3.92 ± 1.96	3.52 ± 2.42	4.47 ± 2.11

**Figure 4:**
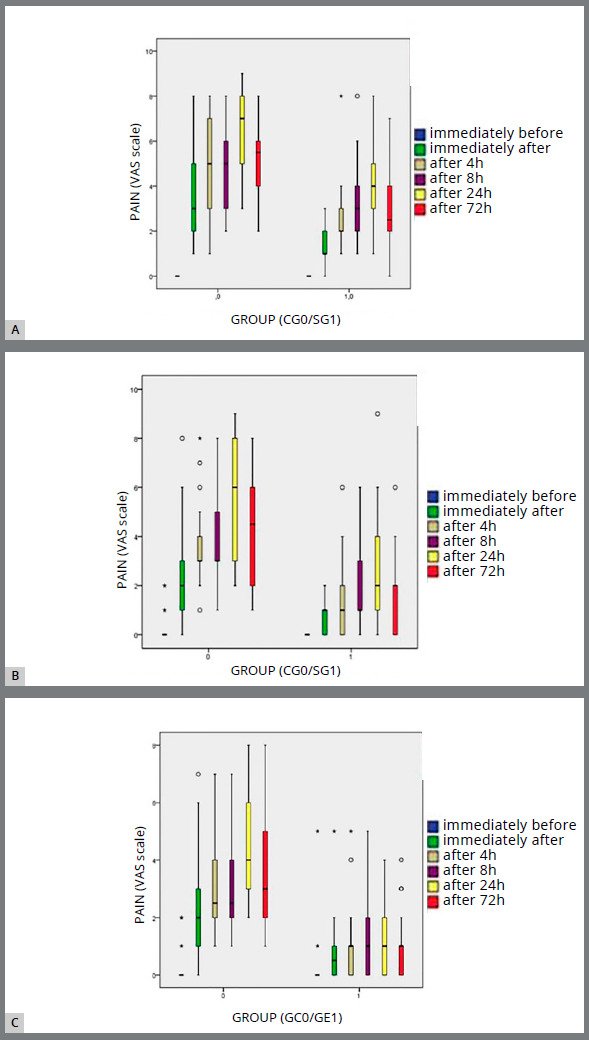
Pain distribution between the SG and CG at: **(**A) the beginning of therapy (T_0_); **(**B) the first adjustment (T_1_); **(**C) the second adjustment (T_2_).

Figure 4 shows the quantitative results related to pain (VAS scale), subdivided by the time interval analyzed: T_0_ (start of therapy), T_1_ (first adjustment), and T_2_ (second adjustment). These results indicate that for both the SG and CG groups, pain appeared immediately after the bonding/adjustments, and tended to increase in the following hours, reaching the highest values ​​after 24 hours. CG patients on average assigned pain values ​​greater than those of the SG for all the time intervals considered (Fig 4).

Table 3 shows the pain severity for both groups in the three therapeutic intervals considered (T_0_, T_1_ and T_2_), divided for each time moment analyzed: immediately before, immediately after, after 4h, after 8h, after 24h, and after 72h. 

**Table 3:** Pain severity in the SG and CG groups, in the three time intervals considered (T_0_, T_1_ and T_2_), divided by time moment.

	AURICULOTHERAPY (SG) Patients: n (%)					NO AURICULOTHERAPY (CG) Patients: n (%)				
Immediately before										
	No pain	Mild	Medium	Severe	Very severe	No pain	Mild	Medium	Severe	Very severe
T_0_*	18 (100)	0	0	0	0	18 (100)	0	0	0	0
T_1_*	18 (100)	0	0	0	0	14 (77.8)	4 (22.2)	0	0	0
T_2_	16 (88.9)	1 (5.5)	1 (5.5)	0	0	15 (83.3)	3 (16.7)	0	0	0
Immediately after										
	No pain	Mild	Medium	Severe	Very severe	No pain	Mild	Medium	Severe	Very severe
T_0_**	2 (11.11)	15 (83.3)	1 (5.5)	0	0	0	7 (38.9)	7 (38.9)	1 (5.5)	3 (16.7)
T_1_*	7 (38.9)	11 (61.1)	0	0	0	1 (5.5)	9 (50)	5 (27.8)	2 (11.11)	1 (5.5)
T_2_	9 (50)	8 (44.4)	1 (5.5)	0	0	2 (11.11)	10 (55.5)	3 (16.7)	3 (16.7)	0
After 4h										
	No pain	Mild	Medium	Severe	Very severe	No pain	Mild	Medium	Severe	Very severe
T_0_*	0	10 (55.5)	7 (38.9)	0	1 (5.5)	0	2 (11.11)	9 (50)	5 (27.8)	2 (11.11)
T_1_*	5 (27.8)	9 (50)	3 (16.7)	1 (5.5)	0	0	4 (22.2)	11 (61.1)	2 (11.11)	1 (5.5)
T_2_*	8 (44.4)	8 (44.4)	2 (11.11)	0	0	0	9 (50)	6 (33.3)	3 (16.7)	0
After 8h										
	No pain	Mild	Medium	Severe	Very severe	No pain	Mild	Medium	Severe	Very severe
T_0_	0	8 (44.4)	8 (44.4)	1 (5.5)	1 (5.5)	0	1 (5.5)	9 (50)	6 (33.3)	2 (11.11)
T_1_*	4 (22.2)	9 (50)	4 (22.2)	1 (5.5)	0	0	4 (22.2)	11 (61.1)	1 (5.5)	2 (11.11)
T_2_*	5 (27.8)	11 (61.1)	2 (11.11)	0	0	0	9 (50)	6 (33.3)	3 (16.7)	0
After 24h										
	No pain	Mild	Medium	Severe	Very severe	No pain	Mild	Medium	Severe	Very severe
T_0_**	0	4 (22.2)	11 (61.1)	1 (5.5)	2 (11.11)	0	0	5 (27.8)	5 (27.8)	8 (44.4)
T_1_*	4 (22.2)	6 (33.3)	6 (33.3)	1 (5.5)	1 (5.5)	0	3 (16.7)	5 (27.8)	3 (16.7)	7 (38.9)
T_2_**	5 (27.8)	10 (55.5)	3 (16.7)	0	0	0	3 (16.7)	9 (50)	3 (16.7)	3 (16.7)
After 72h										
	No pain	Mild	Medium	Severe	Very severe	No pain	Mild	Medium	Severe	Very severe
T_0_*	1 (5.5)	8 (44.4)	6 (33.3)	3 (16.7)	0	0	1 (5.5)	8 (44.4)	7 (38.9)	2 (11.11)
T_1_**	6 (33.3)	8 (44.4)	3 (16.7)	1 (5.5)	0	0	5 (27.8)	8 (44.4)	3 (16.7)	2 (11.11)
T_0_**	8 (44.4)	7 (38.9)	3 (16.7)	0	0	0	7 (38.9)	8 (44.4)	2 (11.11)	1 (5.5)

The Student’s *t*-test was significant in the comparative analysis of pain between the SG and CG for almost all time moments considered, at both the start of therapy and the following two adjustments (T_0_, T_1_ and T_2_) (Table 3).

The Chi-square test did not show significant differences in pain perception for any of the following parameters: age, gender, malocclusion, or treated dental arch (*p*> 0.05). The only exception was TM “after 24 h” in T_0_, where gender was found to be significant (*p*= 0.042). 

## DISCUSSION

Pain is a frequent result of dental procedures, including orthodontic treatments, and is characterized by extreme individual variability. Studies analyzing the evolution of pain in patients with fixed orthodontic appliance show that orthodontic pain arises after application of orthodontic force, with a spike after 12-24 hours and a progressive decrease in the following hours, and can persist for 5-7 days after the force application.^3^ ^,^ ^22^ ^,^ ^23^ The results of this study confirm that evidence: pain occurs, on average, immediately after orthodontic appliance adjustment and progressively increases in the following hours; patients belonging to both study groups indicated higher pain values ​​in the period “after 24h”*,* and these values decreased in the next time interval analyzed.

A non-linear relationship has been established among pain perception in response to orthodontic force and age, gender, and psychological state. Though several studies have reported that adult patients perceive more pain than younger patients, it is difficult to generalize conclusions concerning age-related pain prevalence and characteristics in Orthodontics, especially due to the different therapeutic approaches performed on patients of different ages.^23^ Our analysis also found no significant age-related pain variation in either the SG or CG groups.

Differences in pain perception according to sex and gender have long been debated. Genetic, molecular, physiological, and psychosocial factors contribute to pain elaboration and influence pain perception in separate ways in men and women. Females have a greater prevalence of many clinical pain conditions, and it is believed that they are more sensitive to pain than males.^24^ However, conflicting results regarding male and female pain perception during fixed appliance treatment have emerged. Some studies show no gender differences in reported orthodontic pain with respect to pain threshold.^23^ Only two studies have found that girls report more discomfort/pain and ulceration than boys during fixed orthodontic treatment.^3^ ^,^ ^25^ In this study, male patients seemed to experience more pain, on average, than female patients, but gender was still not found to be a significant factor in determining the onset and intensity of pain.

Other factors were not evaluated in this study, and this can be considered a limitation of the research, since personal and psychological factors can affect the perception of pain. Ideally, sham auriculotherapy (a bandage fixed on the same acupoints, but without the use of *Vaccaria* seeds) should also have been applied in this study. However, this preliminary research aimed to evaluate the potential efficacy of auriculotherapy in the management of orthodontic pain, despite the lack of evidence of its previous use in this specific area; it was therefore considered appropriate to proceed without the sham group. Further studies should aim to improve upon this point by addressing such factors.

The study was carried out by applying a particular acupuncture method: auriculotherapy (AT), which involves the application of *Vaccaria seeds* to specific auricular acupoints. This technique, unlike other acupuncture methods, does not involve the insertion of needles and therefore offers the additional advantage of being well-received by patients, including pediatric subjects. Moreover, it was effective in treating various types of pain, both acute and chronic.^19^ ^,^ ^20^ ^,^ ^26^ A recent review from Vieira et al.^26^ has shown auriculotherapy to have a positive effect when paired with conventional treatments of chronic and acute pain. Iunes et al.^19^ investigated the efficacy of auriculotherapy in a group of TMJ dysfunctional patients, demonstrating auriculotherapy to be significantly effective in reducing pain in the temporal and TMJ areas. A meta-analysis from Yeh et al.^20^ established that auriculotherapy provides significant pain relief when compared to a sham or control group. It has also been demonstrated that auricular acupressure is more effective than auricular acupuncture.^20^ This evidence of the efficacy of auriculotherapy in pain management supports the application of AT in orthodontic pain management. 

Considering the high number of patients reporting discomfort or pain during orthodontic therapies, several analgesic methods have been studied. Most existing studies propose the administration of non-steroidal anti-inflammatory drugs (NSAIDs)*,* which are effective in reducing pain, but may limit the extent of dental movement during therapy.^2^ ^,^ ^4^ ^,^ ^27^ Satisfying results in pain reduction have been found in studies concerning Low-level laser therapy (LLLT)*,* although its use is poorly documented.^7^ ^,^ ^28^ Only a few studies have investigated acupuncture to treat orthodontic pain and none of them studied the application or effectiveness of auriculotherapy. Jia et al.^29^ studied the clinical efficacy of transcutaneous electrical acupoint stimulation (TEAS) for orthodontic toothaches through the use of three acupoints: *Juliao* (ST3), *Jiachengjiang* (Extra), and the auricular point *Ya* (LO1). In this study, the pain scores of the TEAS group were lower than those in the two control groups. An animal study by the same authors showed satisfactory results regarding the therapeutic and preventive effects of TEAS on rabbits with orthodontic toothaches.^17^ To manage post-adjustment orthodontic pain, Vachiramon and Wang^30^ proposed the use of just one acupoint, *Hegu* (LI4), stimulated by needles or simple acupressure. Finally, Boleta et al.^18^ analyzed patients’ pain levels during the second quarter of fixed orthodontic therapy. They applied a treatment of somatic acupuncture, using two acupoints, *Hegu* (LI 4) and *Jiache* (ST 6), and found a statistically significant reduction in pain level indexes, both for men and women, when acupuncture therapy was performed before the orthodontic adjustment. 

These studies highlight the effectiveness of acupuncture in the treatment of pain during fixed orthodontic therapy; the results obtained from the application of auriculotherapy in this study confirm such findings. From the moment of the application of orthodontic force, the study group undergoing auriculotherapy perceived lower pain values than the control group, both at the beginning of therapy (T_0_) and in the two consecutive months of treatment (T_1_ and T_2_). The results show this difference in perceived pain for all the time moments considered (immediately before, immediately after, after 4h, after 8h, after 24h, and after 72h), with a statistically significant difference between average values of perceived pain for most time moments considered (Table 3). 

The therapeutic auriculotherapy protocol proposed in this study is versatile, easy to apply, minimally invasive, and low-cost. Acupuncture procedures, including auriculotherapy, do not produce side effects and can be safely applied by qualified acupuncturists. This is an important feature, especially given the extreme heterogeneity of orthodontic patients, high percentage of pediatric subjects, and growing demand from patients to limit the use of medication. 

## CONCLUSION

Auriculotherapy seems to be effective in pain management for patients undergoing fixed orthodontic treatment. Despite the limitations of this study, we can consider auriculotherapy a valid analgesic alternative in the treatment of orthodontic pain. Further studies must be conducted to confirm the results obtained. 
